# Evaluation of cephalometric points in midface bone lengthening with the use of a rigid external device in syndromic craniosynostosis patients

**DOI:** 10.1016/S1808-8694(15)30658-3

**Published:** 2015-10-19

**Authors:** Daniel Santos Corrêa Lima, Nivaldo Alonso, Paulo Roberto Pelúcio Câmara, Dov Charles Goldenberg

**Affiliations:** 1 Master's degree, USP Medical School. Substitute assistant professor, Plastic Surgery Discipline, Medical School of the Universidade Federal da Bahia. In charge of the craniomaxillofacial surgery sector; 2 Livre-docente (habilitation) professor, USP Medical School. In charge of the craniomaxillofacial surgery sector, Plastic Surgery and Burns Division, Hospital das Clínicas da FMUSP; 3 Master's degree in orthodontics. Orthodontist of the craniomaxillofacial surgery sector, Plastic Surgery and Burns Division, Hospital das Clínicas da FMUSP; 4 Doctorate, USP Medical School. Assistant physician, craniomaxillofacial surgery sector, Plastic Surgery and Burns Division, Hospital das Clínicas da FMUSP. Plastic Surgery and Burns Division, Hospital das Clínicas da FMUSP

**Keywords:** bone lengthening, craniosynostosis, external fixators, facial bones, distraction osteogenesis

## Abstract

Distraction osteogenesis has been extensively used to correct severe midface hypoplasia in syndromic craniosynostosis patients. However few studies have reported midface distraction outcomes through cephalometric evaluation.

**Aim:**

The purpose of the present study was to evaluate outcomes with midface distraction rigid external device (RED) in patients with syndromic craniosynostosis, in terms of quantity of bone lengthening, skeletal stability and facial growth.

**Materials and methods:**

Eleven patients were retrospectively evaluated in this study. Cephalometrics was carried out through three teleradiographies from each patient (T1 -before surgery; T2- immediate postop, rigth after distractor removal; T3 - late postop, obtained with a minimal interval of 12 months after surgery).

**Results:**

Significant midface advancement was achieved with the procedure. The rate of horizontal relapse was minimal. We noticed a clear vertical facial growth, contrary to what was seen in the horizontal aspect, when there was a mild posterior relapse and no growth evidence.

**Conclusion:**

Cephalometric evaluation showed adequate results in midface bone lengthening with rigid external distractor.

## INTRODUCTION

Midface advancement to attain an adequate facial appearance is the main feature in the treatment of facial dysostoses. The critical point for successful therapy is the ability to advance and maintain the advanced bone segments in an anatomically normal position.^1^

In 1950, Gilles and Harrison^2^ performed the first midface advancement surgery to correct skeletal hypoplasia in a patient with syndromic craniosynostosis; this was a technically difficult and lengthy procedure, which led to it being abandoned after this first case.^3^ Tessier^4^,^5^ described an osteotomy technique according to Le Fort III type fractures; this procedure has become the standard technique for the treatment of craniofaciosynostosis. In 1978, Ortiz-Monasterio et al.^6^ published the monobloc frontofacial advancement technique, in which not only the midface but also the orbits and the frontal region are advanced in one piece. This technique, however, has been associated with a high infection rate due to exposure of the ethmoidal sinuses and nasal cavities to the intracranial space;^3^,^7^, ^8^, ^9^, ^10^, ^11^, ^12^ other important complications including cerebrospinal fluid fistulas and frontal bone necrosis.^3^

Aside from the mortality associated with major surgery, the amount of advancement and the skeletal stability that can be attained in these conventional procedures is limited mostly because of soft tissue resistance to bone movement.

Distraction osteogenesis has gained popularity in the treatment of facial skeletal deformities as a method for overcoming these limitations; it has become the standard technique for the treatment of facial bone hypoplasia in patients with syndromic craniosynostosis.

Few studies, however, have assessed the results of midface distraction osteogenesis with cephalometry; this method is more appropriate for evaluating the results of procedures that involve osteotomies and bone segment movements following osteotomies for correcting facial skeletal deformities.

## OBJECTIVE

The purpose of this study was to assess the results of midface advancement associated with a rigid external device in patients with syndromic craniosynostosis by analyzing cephalometric reference points.

## MATERIAL AND METHOD

The Research Ethics Committee of the institution approved this study. An observational longitudinal retrospective descriptive study was made of data gathered from files and assessments of radiological exams (lateral tele-radiography of the face) done pre- and postoperatively of patients with syndromic craniosynostosis undergoing distraction osteogenesis with the Le Fort III type osteotomy (DOLF) or distraction osteogenesis with monobloc frontofacial osteotomy (DOM) with a rigid external device (RED). The sample comprised 11 patients with syndromic craniofaciosynostosis seen at the Craniomaxillofacial Surgery Sector of the Plastic Surgery and Burn Division of the Hospital das Clinicas, Sao Paulo University Medical School; these patients were treated surgically for the correction of midface hypoplasia from 2002 to 2006. The following types of syndromic craniofaciosynostosis were diagnosed in these patients: Crouzon's syndrome (craniofacial dysostosis), n=6; Apert's syndrome (type I acrocephalosyndactyly), n=4; Saethre-Chotzen's syndrome (type III acrocephalosyndactyly), n=1.

There were three male and eight female patients.

The age at which patients were operated ranged from 5 to 17 years (mean 9 years). The follow-up time ranged from 12 to 39 months (mean 17 months).

Indications for surgery were significant facial alterations typical of craniofacial dysostoses in the sample patients. In all cases there was midface skeletal hypoplasia and Angle class III malocclusion. Exorbitism was present in all cases; in three patients, there was significant ocular displacement. Surgery consisted of a Le Fort III osteotomy or monobloc frontofacial osteotomy, according to each case, and placement of an external rigid distraction device (rigid external distraction system or RED, KLS Martin, Germany). Activation of the distraction device (1.0 mm per day) was initiated after a 5-day latency period until occlusion was overcorrected to attain an Angle class II relation; an adequate relation between the orbit and its contents; and adequate frontal projection, in cases where monobloc frontofacial osteotomy was done.

The external rigid device remained in place as a fixation and retention appliance for six to eight weeks after the desired advancement of midface bone segments was attained.

**Figure 1:**
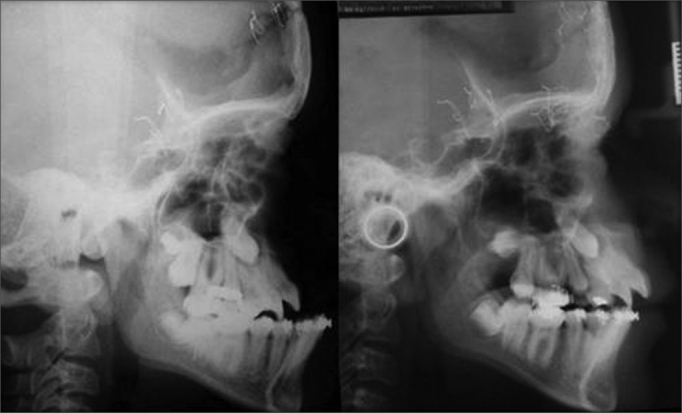
Lateral teleradiograph of a patient undergoing a Le Fort III type osteotomy followed by midface distraction osteogenesis with a rigid external device (RED). Left: preoperative. Right: early postoperative.

Cephalograms were obtained from three lateral teleradiographs of the face of each patient (Fig. 1).

1st teleradiograph: done preoperatively.

2^a^ teleradiograph: done in the early postoperative period soon after removing the device at the end of bone consolidation.

3^a^ teleradiograph: done at least 12 months after surgery.

The same researcher performed all cephalometric tracings. Thirty-three tracings were obtained. The cephalograms were done using a negatoscope; 18 × 24 cm (0.07 mm thick) acetate paper for cephalometric tracings; a cephalometric ruler; a 0.5mm lead pencil; black, red and blue 0.5 thickness graphite; adhesive tape; a white soft eraser.

The anatomical landmarks of the anterior portion of the cranial base were marked directly on each teleradiograph with black 0.5 mm graphite, according to the total structural cephalometric superposition method. Thus, anatomical landmarks such as the anterior border of the sella turcica, the optic canal, the superior aspect of the sphenoid bone body, the sphenoethmoidal suture, and the horizontal portion of the inner cortex of the frontal bone, were used as superposition parameters in teleradiographs. The three teleradiographs of each patient (T1, T2 and T3) were superimposed to yield three cephalometric tracings on the same acetate paper.

**Figure 2:**
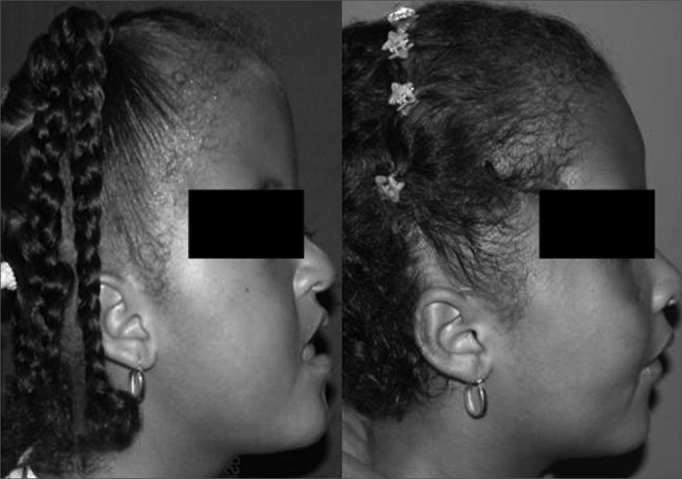
Patient undergoing monobloc frontofacial osteotomy and facial bone lengthening with a rigid external distractor. Left: preoperative. Right: one-year postoperative.

1) The first teleradiograph (T1: preoperative) was placed on the negatoscope; its left lateral margin was fixed to the surface of the negatoscope with adhesive tape.
2) The second teleradiograph (T2: early postoperative) was superimposed on T1, based on the aforementioned anatomical parameters. T2 was fixed to the surface of the negatoscope by its right lateral margin.
3) Finally, T2 was moved away, and the third teleradiograph (T3: late postoperative) was superimposed on T1; it was fixed by its upper margin.
4) After fixing T1, T2 and T3, T1 was moved away to check whether T2 and T3 were perfectly superimposed. Thus, all three teleradiographs were superimposed. If necessary, adjustments were made in the position of the teleradiographs until all three were perfectly superimposed.
5) Acetate paper was then fixed along it lower margin. Tracings were done by placing the paper over each teleradiograph in turn. Preoperative tracings were made in black ink. Postoperative tracings were made in red ink (early postoperative) and blue (late postoperative). The patient's name and date of the exam were recorded on the upper right corner of each teleradiograph.
6) The natural position of each patient's head was established based on observations of photographs and teleradiographs. A vertical reference line (true vertical line) was then marked (Figure 1, Figure 2).

Each patient's acetate paper with the three tracings was digitized using a 300 dpi Scanjet 3670 (Hewlett-Packard Development Company, HP) scanner.

**Figure 3:**
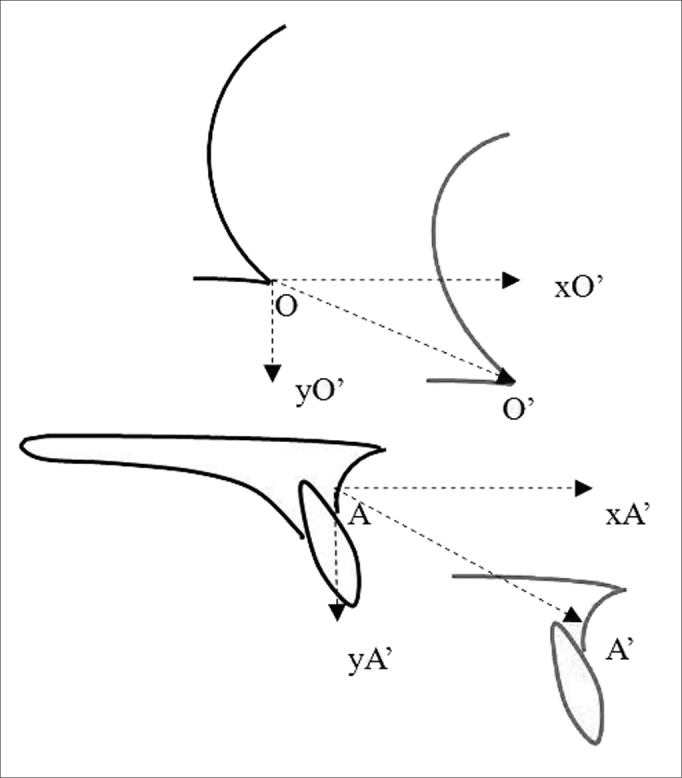
Representation of mean distances between preoperative tracings (in black) and early postoperative tracings (in red). A: point A in the preoperative tracing; A': point A no early postoperative tracing; xA': projection of point A' on the horizontal axis drawn from point A; yA': projection of point A' on the vertical axis drawn from point A; O: orbit point on the early postoperative tracing; O': orbit point on the early postoperative tracing; xO': projection of point O' on the horizontal axis drawn from point O; yO': projection of point O' on the vertical axis drawn from point O. A-A': distance between points A and A' = resulting displacement measured from point A, on the movement vector; A-xA': distance between point A and the projection of point A' on the horizontal axis = horizontal advancement measured from point A. A-yA': distance between point A and projection of point A' on the vertical axis = vertical displacement measured from point A; O-O': distance between points O e O' = resulting displacement measured from point O, on the movement vector; O-xO': distance between point O and projection of point O' on the horizontal axis = vertical displacement measured from point O; O-yO': distance between point O and the projection of point O' on the vertical axis = vertical displacement measured from point O.

The following cephalometric reference points were used as measuring parameters: point A (most posterior point of the concavity of the anterior surface of the maxillary alveolar process); point O (orbit point - intersection point between the border of the orbit floor and the lateral orbit border). A' and O' were the reference points in early postoperative tracings, xA' and xO' were their horizontal extensions, and yA' and yO' were their vertical extensions. A” and O” were the reference points in late postoperative tracings, xA” and xO”, and yA” and yO” were their horizontal and vertical extensions. Study variables were the distance between reference points in the three tracings:

Distances measured between pre- and early postoperative tracings were: a) resulting bone length gain along the movement vector (distances A-A' and O-O'), b) horizontal advancement (distances A-xA' and O-xO'), c) vertical displacement (distances A-yA') (Fig. 3).

**Figure 4:**
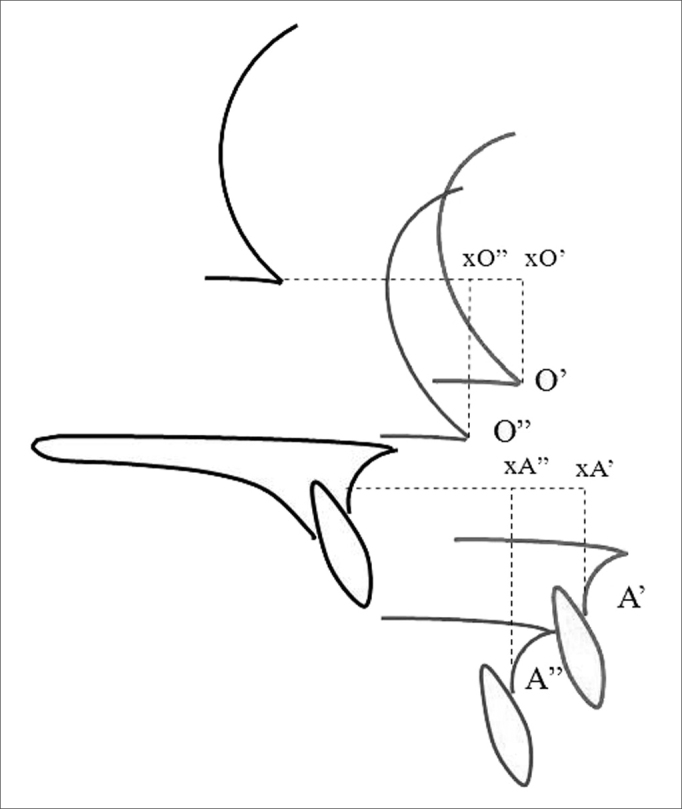
Representation of late horizontal repositioning measures: distances measured from reference points between early postoperative tracings (in red) and late postoperative tracings (in blue). A': point A on the early postoperative tracing; xA': projection of point A' on the horizontal axis drawn from point A; A”: point A on the late postoperative tracing; xA”: projection of point A” on the horizontal axis drawn from point A on the late postoperative tracing; O': point O on the early postoperative tracing; xO': projection of point O' on the horizontal axis drawn from point O; O”: point O on the late postoperative tracing; xO”: projection of point O” on the horizontal axis drawn from point O on the late postoperative tracing. Distance xA'-xA”: amount of late posterior horizontal repositioning for point A. Distance xO'-xO”: amount of late posterior horizontal repositioning for point O.

**Figure 5:**
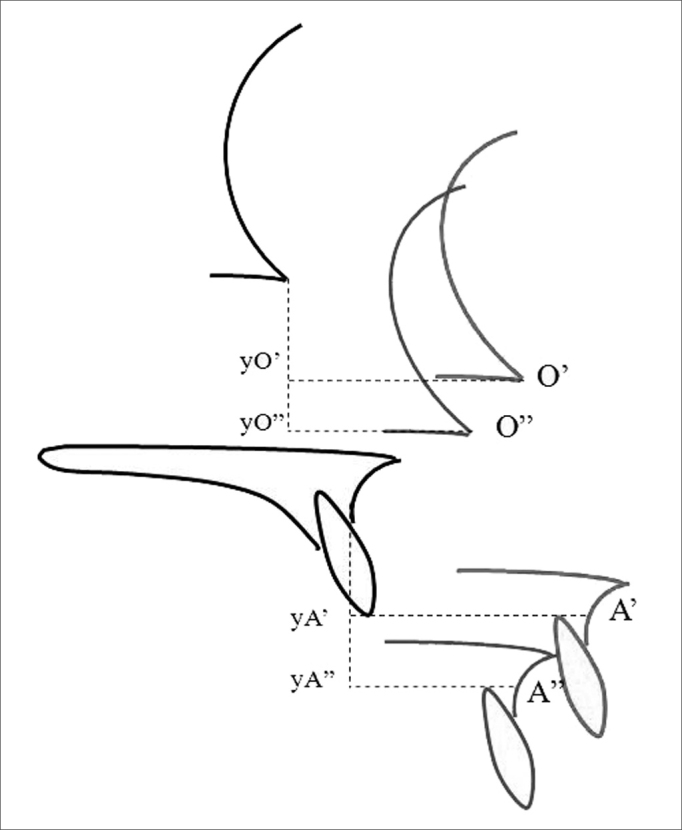
Representation of late vertical repositioning measures: distances measured for reference points between early postoperative tracings (in red) and late postoperative tracings (in blue). A': point A on the early postoperative tracing; yA': projection of point A' on the vertical axis drawn from point A; A”: point A on the late postoperative tracing; yA”: projection of point A” on the vertical axis drawn from point A on the late postoperative tracing; O': point O no early postoperative tracing; yO': projection of point O' on the vertical axis drawn from point O; O”: point O no late postoperative tracing; yO”: projection of point O” on the vertical axis drawn from point O on the late postoperative tracing. Distance yA'-yA”: amount of late vertical repositioning for point A. Distance yO'-yO”: amount of late vertical repositioning for point O.

Distances measured between pre- and early postoperative tracings were: a) amount of late posterior repositioning (distances xA'-xA” and xO'-xO”) (Fig. 4); b) amount of late vertical repositioning (distances yA'-yA” and yO'-yO”) (Fig. 5).

The appropriate descriptive statistics were calculated for quantitative variables: the mean, the standard deviation, the coefficient of variability, and the interquartile range and mean. The Shapiro-Wilk test was applied to test for a normal distribution in the association among variables. Pearson's correlation was applied to test the relationship to the theoretical normal distribution. Spearman's correlation was applied when the distribution was not normal. The significance level in this study was 5%.

## RESULTS

For the horizontal advancement from point A (variable A-xA'), the resulting mean advancement was 10.45mm ± 6.8mm (mean and standard deviation). The variation coefficient of this measure was 65.14%; the median was 11.2mm, and the interquartile range was 7.55mm. For the horizontal advancement from point O (variable O-xO'), the resulting mean advancement was 9.26 ± 3.82mm; the variation coefficient was 41.34%. The median for this variable was 9.4mm; the interquartile range was 3.55mm (Table 1).

**Table 1:** Descriptive statistics for the study variables, found from measurements of point A and the orbit point, in preoperative and early postoperative tracings

Variable	Minimum	Maximum	Mean	SD	VC%	Median	IQR
A-xA′	1,2	25,0	10,45	6,80	65,14	11,2	7,55
A-yA′	-4,4	10,3	3,18	4,89	153,8	2,6	8,3
A-A′	4,9	25,0	12,41	5,65	45,54	11,2	5,5
O-xO′	2,0	15,7	9,26	3,82	41,34	9,4	3,35
O-yO′	-3,1	7,8	2,39	3,71	155,5	2,7	5,4
O-O′	6,6	17,5	10,33	3,42	33,16	9,5	4,25

A-xA': horizontal advancement measured from point A; A-yA': vertical displacement measured from point A; A-A': resulting movement vector measured from point A; O-.xO': horizontal advancement measured from point O; O-yO': vertical displacement measured from point O; O-O': resulting movement vector measured from point O. Minimum: minimum variable value; maximum: maximum variable value; median: mean variable value; SD: standard deviation; VC: variation coefficient; IQR: interquartile range.

Variables A-yA' and O-yO' (vertical displacement from A and O) were negative or positive, as the vertical displacements occurred inferiorly in most cases (positive values) or superiorly in some cases (negative values). Values for the vertical displacement for point A (variable A-yA') were expressed as the mean and standard deviation (3.18mm ± 4.89mm). The variation coefficient was 153.8%. The median was 2.6mm and the interquartile range was 8.3mm. The mean vertical displacement for point O (variable O-yO') was 2.39mm ± 3.71mm. The variation coefficient was 155.5%. The median was 2.7mm and the interquartile range was 5.4mm (Table 1). The wide dispersion for these two variables, as shown in the variation coefficient, was due to a significant variability in the amount of vertical movement seen in the sample tracings. This variation was made evident because the study included positive and negative results. An inferior displacement predominated over a superior displacement, as shown by the positive mean and median values.

Variables A-A' and O-O' (resulting from bone movement, measured from points A and O) were upwards or downwards, depending on their vertical component. An ascending vector was seen in four cases, and a descending vector was seen in seven cases. The mean (and standard deviation) of variable A-A' was 12.41 ± 5.65mm; the variation coefficient was 45.54%. The median was 11.2mm and the interquartile range was 5.5mm. The mean (and standard deviation) of variable O-O' was 10.33 ± 3.42mm; the variation coefficient was 33.16%. The median was 9.5mm and the interquartile range was 4.25mm (Table 1).

Variables showing the horizontal (A-xA'), vertical (A-yA') and resulting (A-A') movement of point A were compared to the variables showing the same movement for the orbit point (O-xO', O-yO' and O-O'). The correlation test was applied for this comparison. There was a positive correlation for the variables A-xA' and O-xO', with a high association rate among variables (r = 0.82); the statistical significance (p) was 0.002. Correlation among the variables A-yA' and O-yO' was also positive; the correlation coefficient (r = 0.89) showed that the variables were highly associated, with a statistical significance (p) < 0.0001. There was a positive correlation between the variables ΔA and ΔO, expressed by its correlation coefficient (r = 0.72); the statistical significance (p) was < 0.0001 (Table 2). These results, which show a significant association among variables, demonstrate that the magnitude of movements measured from point A was uniform compared to the same movements measured from the orbit point.

**Table 2:** Correlation among variables found in measurements from point A and the orbit point, in preoperative and early postoperative tracings.

Variables		r	P
A-xA′	O-xO′	0,82	0,002*
A-yA′	O-yO′	0,89	0,0001*
A-A	O-O′	0,72	0,0001*

Spearman's correlation coefficient (r) for the variables A-xA′ and O-xO′; A-yA′ and O-yO′; A-A′ and O-O′. A-xA': horizontal advancement measured from point A; A-yA': vertical displacement measured from point A; A-A': resulting movement vector measured from point A; O-xO': horizontal advancement measured from point O; O-yO': vertical displacement measured from point O; O-O': resulting movement vector measured from point O. (p: descriptive level test of r being equal to zero hypothesis);

* statistically significant.

Late results were gathered from measurements comparing the early and late postoperative measurements.

Late posterior horizontal repositioning (variables xA'-xA” and xO'-xO”) was the loss of results, reflecting the stability of skeletal advancement. Late posterior horizontal repositioning for the variable xA'-xA” was −0.96mm ± 0.72mm (mean and standard deviation). The variation coefficient was 75.02%. The median was 0.6mm, with an interquartile range of 1.0mm. The value for the variable xO'-xO” was −0.85mm ± 1.09mm (mean and standard deviation). The variation coefficient was 121.7%. The median was −1.0mm and the interquartile range was 0.95mm (Table 3). These numbers reflect the stability of results, since late posterior repositioning, or loss of results, was less than 1.0mm in most cases for measurements done from both cephalometric points (points A e O). A comparison of the magnitude of posterior horizontal repositioning horizontal posterior with the measured horizontal advancement, verified by measurements comparing A and orbit points between pre- and late postoperative tracings (A-xA' and O-xO'), revealed a 5.3% loss of results rate for point A and a 10.6% loss of results for the orbit point.

**Table 3:** Descriptive statistics for measurements made from point A and the orbit point in early and late postoperative tracings.

Variable	Minimum	Maximum	Mean	SD	VC %	Median	IQR
xA'-xA″	-0,3	-2,6	-0,96	0,73	75,02	-0,6	1,0
yA'-yA″	0,6	6,7	3,45	1,94	56,27	3,4	2,65
xO'-xO″	-0,3	-1,9	-0,85	1,03	121,70	-1,0	0,95
yO'-yO″	1,6	5,4	2,92	1,25	42,85	2,3	1,95

xA′-xA″: late horizontal repositioning measured from point A; yA′-yA″: late vertical repositioning measured from point A; xO′-xO″: late horizontal repositioning measured from point O; yO′-yO″: late vertical repositioning measured from point O. Minimum: minimum value for the variable; maximum: maximum value for the variable; median: median value for the variable; SD: standard deviation; VC: variation coefficient; IQR: interquartile range.

Late vertical repositioning (variables yA'-yA” and yO'-yO”) was the facial growth along the vertical axis. The vertical position of the cephalometric points (A and orbit points) was more inferior in late postoperative compared to early postoperative tracings in all cephalograms. The value for the variable yA'-yA” was 3.45mm ± 1.94mm (mean and standard deviation). The variation coefficient was 56.27%. The median was 3.4mm; the interquartile range was 2.65mm. The mean value for the variable yO'-yO” was 2.93mm ± 1.25mm; the variation coefficient was 42.85%. The median was 2.3mm, and the interquartile range was 1.95mm (Table 3).

## DISCUSSION

Many studies have shown that conventional procedures for treating facial deformities in syndromic craniosynostosis patients yield limited results. Conventional Le Fort III osteotomy and advancement of the midface (CLFO) and conventional monobloc frontofacial advancement (CMFA) result in little bone advancement, especially because of soft tissue resistance.

Cases treated conventionally show a mean facial advancement ranging from 6 to 17mm; correction in most cases is about 10mm, according to Meling et al.^7^ Studies by Firmin et al.,^13^ McCarthy et al.,^14^ Bachmeyer et al.,^15^,^16^ Kaban et al.,^17^ Kreiborg and Aduss18 and Ousterhout et al.^19^ have corroborated these numbers. However, anteroposterior orbitary and midface deficiency in syndromic craniosynostosis patients is considerably larger, as shown in some studies that underline the need for advancement up to 24mm;^20^, ^21^, ^22^ this value exceeds the amount of bone displacement attainable with conventional procedures without any risks of therapeutic failure.

After distraction osteogenesis was added to the treatment of facial deformities in syndromic craniosynostosis, attainable advancement has become consistently larger, compared to conventional advancement results. In 2001, Fearon^23^ published a study of 16 syndromic craniosynostosis patients divided into two groups. The first group (n=7) underwent the conventional Le Fort III type osteotomy (CLFO), and the second group underwent a distraction osteogenesis Le Fort III type osteotomy (DOLF) with a rigid external device (RED). Mean advancement in the first group was 5.1mm, with a maximum 16.0mm advancement, as measured from point A. Mean bone lengthening from point A in the second group was 16mm, reaching a maximum 35.0mm increase in length. A comparison of the advancement attained by each procedure in each group revealed that the differences were statistically significant (p<0.005). More recently, Iannetti et al.^24^ published a study comparing a group of patients that underwent conventional Le Fort III type surgery (n=5) with a group that underwent facial bone lengthening by using an internal distraction device (n=10). Maximum advancement in the first group was 14.0mm (mean 8.6mm); maximum length increase in the second group was 22.0mm (mean 13.9mm).

Advancement attained with midface distraction osteogenesis associated with the Le Fort III osteotomy has been demonstrated in other series. Toth et al.^22^ published the result of midface bone lengthening by using an internal device in 15 patients. The mean advancement was 19.7mm, with a maximum advancement of 30.0mm. Holmes et al.^25^ attained a mean 10.0mm horizontal advancement and a maximum 16mm advancement by associating the Le Fort III procedure with an internal distractor, in eight patients. Denny et al.^26^ showed a 14.0mm mean advancement (maximum 21.0mm) as a result of midface distraction with a Le Fort III procedure with an internal distractor.

Gosain et al.'s^27^ series described a 14.85mm mean advancement (maximum 26.0mm). In this study, a Le Fort III osteotomy with distraction using an internal device was applied in most cases; an external distractor was used in two cases. In a study assessing the results of associating fronto-orbital advancement and midface distraction osteogenesis with an external device and Le Fort III in six patients, Kubler et al.^8^ demonstrated a mean 15.8mm advancement (maximum 18.0mm). Tunçbilek et al.^28^ attained a mean 15.3mm advancement in three patients (maximum 18mm) by associating an external device and the Le Fort III. Fearon^1^ assessed the results in a series of 23 patients in which the mean advancement was 16.70mm. Shetye et al.^29^ published a recent paper 15 patients in whom midface distraction was done with an external distractor (RED); the mean advancement from point A was 15.85mm.

Our study on midface advancement using RED and the Le Fort III procedure resulted in a mean advancement of 8.29mm (median 7.55mm). These values, although lower than those reported in the literature, were attained in a group of only four patients. The minimum (4.9mm) and maximum (15.7mm) values in this series, compared to the literature, shows the impact of a small number of cases on the mean. The mean was 13.9mm in Iannetti et al.'s^24^ series, for instance; advancement was only 5.0mm in three patients and 8.0mm in one of 10 patients. The high mean was due to two cases in which the necessary advancements were 25mm and 35mm. In Holmes et al.'s^25^ paper, four of seven patients had advancements below 10mm; the lowest value in this series was 6mm. In Gosain et al.'s^27^ results, the minimum advancement was 9mm; three of eight cases had an advancement of 12mm or less. In Denny et al.'s^26^ series, five of ten patients had advancements ranging from 10 to 12mm. In other series, results are presented only as means, without individual case values.^23^,^29^

Published results for distraction osteogenesis with monobloc frontofacial osteotomy (DOM) are fewer than the data available for DOLF. Cohen et al.^30^,^31^ reported a 22 to 30mm advancement with an internal device in DOM. Talisman et al.^32^ attained a 20mm advancement in one case by using an external device fixed by transcutaneous pins to the frontal and zygomatic regions. The numbers such as bone movement in these studies, however, refer to the total activation of the distraction device in millimeters according to the number of turns on the activation screw. These values do not faithfully reveal the true movement of bone structures; results demonstrated by cephalometric analyses are more reliable. Cedars et al.^20^ noted this fact, suggesting that the discrepancy between device activation and true bone movement was due to soft tissue resistance. Furthermore, the increased bone length according to activation of the screw in fact reflects the resulting movement on the vector of the distractor, rather than the horizontal movement itself (horizontal advancement), which composes the resultant value with the vertical component.

Bradley et al.^3^ published the only study of a cephalometric analysis of cases undergoing DOM; in this case, an internal distractor was used. In one group, 12 patients underwent conventional monobloc frontofacial advancement; a second group (n=11) underwent modified monobloc frontofacial advancement; and a third group (n=24) underwent DOM. The resulting horizontal movements measured for each group from point A were 9.1mm; 9.4mm and 12.6mm; more advancement was attained in patients undergoing DOM, which was statistically significant. There is a paucity of knowledge about the cephalometric assessment of midface distraction osteogenesis in monobloc osteotomy operated cases.

Our advancement results were 11.2mm (median) and 10.45mm (mean); the maximum advancement was 25.0mm. The mean was influenced by two cases in which there were technical issues. In the first of these cases, the metal bands coupled to the molars became loose, which affected the length gain process; in this case, advancement was only 2.9mm. In the other case, failure was due to incomplete disjunction; advancement was only 1.2mm.

If these two failed cases are excluded, the mean advancement becomes 12.31mm, similar to Bradley et al's result.^3^

The amount of vertical displacement in our measurements was considerably lower than the horizontal component. Although the skeletal deficiency that is typical of craniofaciosynostosis is three-dimensional, it is expressed mostly in the anteroposterior direction; the bone movement requirement is mostly horizontal. Thus, the main purpose of therapy is to move the bone segments anteriorly. The amount of vertical movement is defined by the midface height deficiency, when present.

Cedars et al.^20^ assessed cases operated using DOLF with an external distractor, attaining a mean 2.0mm of inferior vertical movement for the orbit point, and 3.0mm for point A; the mean advancement was 15mm. These authors reported ascending vertical movement in only one case. Shetye et al.^29^ reported a 1.06mm inferior vertical movement and a 15.85mm horizontal advancement in a series operated with DOLF with an external distractor. Other studies do not contain data on the midface vertical distraction component in the treatment of craniofaciosynostosis.

Our results show that the horizontal movement predominated, which agrees with the abovementioned studies. The vertical component predominated only when there were technical issues.

Skeletal stability refers to maintenance of bone lengthening. This factor has also been suggested as an advantage of distraction osteogenesis over conventional procedures. Often major advancement may be attained technically, but instability in these cases greatly increases the risk of lost results. Traction by soft tissues and bone graft resorption due to lack of contact or intense compression are causes of lost results or even recurrences, which characterizes treatment failure. Stable results, even with major bone movement, have become possible with the advent of distraction osteogenesis; this method also gradually lengthens soft tissues, thus overcoming their resistance. Improved results also derive from the formation of new orthotopic bone, which is superior to bone grafts in quality. Other advantages of this procedure are lower morbidity and a shorter operative time, since rigid external fixation and bone grafts are unnecessary.^20^

A tendency for lost results in CLFO has been demonstrated in many papers. Tessier^4^ underlined this trend by defending sagittal overcorrection of 6 to 8mm as a preventive measure against recurrence. Bachmayer et al.^15^ found loss of results ranging from 0 to 4.2mm (mean 1.35mm) among 19 patients, which corresponded to a 9.4% rate (ranging from 0 to 32.2%). Freihofer^33^ attributed the loss of results in two of three patients undergoing CLFO to technical issues, such as failure in skeletal fixation, and postoperative complications. Although late retropositioning of the advanced bone segment was not presented numerically (cephalometry), both cases were classified as “clinically unacceptable recurrence,” which demonstrated the magnitude of lost results.

Kaban et al.^34^ reported significant loss of results in seven of 19 patients in their CLFO series. David and Sheen^35^ analyzed the results of midface advancement using CLFO and CMFA in 16 patients. In three of these patients, loss of results ranged from 1.0 to 2.0mm. Meazzini et al.^36^ showed that in two or eight patients, CLFO was associated with clinically significant loss of results or relapse; in these cases, posterior repositioning ranged from 3.5 to 4.5mm. In the remaining six cases, maximum loss was 1.0mm. These authors attributed relapses to technical issues in the rigid skeletal fixation.

The stability of advancement attained by midface distraction osteogenesis has in most cases been analyzed only using clinical parameters, although many studies have underlined procedure-related stability.

Few studies have based their assessments on measureable data, such as cephalometry; although this method has its limitations, it is the best tool for verifying the results of bone segment mobilization to correct skeletal and dentoskeletal disproportions of any cause.

Fearon^1^ published the results of a large series of patients that underwent DOLF with a rigid external distractor, in which the mean observed loss of results between the early and late postoperative period up to five years was 0.3mm as measured from point A. Shetye et al.^29^ assessed a group of 15 patients that underwent DOLF with a rigid external distractor, in which mean posterior repositioning between the early and late postoperative period (1 year) was 0.07mm for the orbit point. For the point A, these authors found that the mean gain between the early and late (1 year) postoperative period was 0.81mm, which the authors interpreted as due to anteroposterior maxillary growth. Bradley et al.^3^ (2006) compared the stability between conventional monobloc frontofacial advancement (n=12), the modified frontofacial advancement (n=11), and DOM in a 24-patient group. The mean posterior repositioning, measured from point A, was 5.0mm for the monobloc frontofacial advancement and the modified monobloc frontofacial advancement; in these cases, loss of results were 65% and 45% respectively. Loss of results was 1.0mm in the DOM with internal distractor group (8% loss of results). The difference between the first two groups and the DOM group was statistically significant for the variable.

Facial growth has been widely debated in syndromic craniosynostosis. Studies have diverged in various aspects of this theme.

Few studies have described facial growth patterns in non-operated syndromic craniosynostosis patients. Bachmayer et al.^16^ attempted to predict the expected horizontal (anteroposterior) and vertical facial growth in craniofaciosynostosis based on teleradiographs of 52 non-operated patients with Crouzon's, Apert's and Pfeiffer's syndromes; cephalometric measurements were taken and the data was analyzed cross-sectionally by regression analysis. The calculated mean expected sagittal growth in these patients during 4.5 years was 3.2mm, or 0.7mm/year. The projected vertical growth was 5.4mm in 4.5 years, or 1.2mm/year. Of note is the fact that the measurement parameter for horizontal growth in that study was the distance between point A and the basion. Meazzini et al.^36^ and Kreiborg e Aduss^18^ have shown that the posterior portion of the cranial base in many craniofaciosynostosis patients grows (2 to 5mm growth of the posterior cranial base). The basion, therefore, is not an adequate reference point for such measurements, which may have influenced Bachmayer et al.'s results, wherein the assessment of maxillary growth in the sample could not be evaluated independently.

Meazzini et al.^36^ showed that there was no horizontal growth in seven non-operated patients with Crouzon's and Apert's syndromes during a mean period of 6.2 years. This same study also demonstrated vertical growth of 1 to 6mm as measured from the anterior nasal spine, and of 0 to 2mm for the orbit point during the same period.

Another issue in debate is facial growth following midface advancement. Tessier^4^ raised the hypothesis that a Le Fort III type facial advancement would set facial growth in motion. This hypothesis, however, was rejected based on the findings of many authors that secondary procedures on the midface were required during growth in patients operated at an early age.

On the other hand, some authors have suggested that interventions such as the Le Fort III type osteotomy could impair subsequent facial growth. These theories were based on experimental studies demonstrated inhibited facial growth as a result of interventions on facial sutures in pigs,^37^ scars made in soft tissues during Le Fort I type osteotomies in primates,^38^ and surgical damage to the periosteum of the canine hard palate.^39^

Freihofer^33^ reported three cases of midface advancement in which there was marked inhibition of growth, which was attributed to surgical damage. Bachmayer et al.^16^ found that there was no horizontal growth in a group of patients subjected to CLFO, contrary to what had been expected based on growth estimates from regression analysis of measurements of non-operated patients. However, the cephalometric measurement method and the study design were different in both groups: a cross-sectional cohort study of the non-operated group and a longitudinal study of the operated group. As mentioned, the impression that there is horizontal growth may be due to using the distance from point A to the basion as a measurement.

Kaban et al.^34^ suggested that facial growth was present during development in CLFO-operated patients. These authors found that, in their 33-case series, the final position of the midface three or more years postoperatively was anterior relative to its initial position (immediate postoperative period) in eight patients operated during their growth phase (8 to 12 years); the reference was point A. Of note, however, is the fact that the reference line used for measurements in Kaban et al.'s study was the true horizontal line (a line at a 7° angle with the sella-nasion line), which is an error in the cephalometric method; this angle may be much higher because of verticalization of the anterior cranial base in many cases of syndromic craniosynostosis.^40^ For geometrical reasons, therefore, when using the true horizontal line as a reference, a vertical movement is assumed as horizontal, which may explain the author's impression that there was sagittal growth following CLFO.

Based on the results of a 16-patient series undergoing CMFA and CLFO, with a 2-year follow-up, David and Sheen^35^ suggested that there was facial growth ranging from 1 to 12mm. Again, these results may have been due to errors in the cephalometric method, since these authors used a line at a 130° angle with the sella-basion line as a horizontal reference, which may result in the same type of ambiguity found Kaban et al's study.

Feraon^1^ assessed facial growth behavior in syndromic craniosynostosis patients undergoing DOLF. This study demonstrated lack of sagittal growth in 23 patients monitored for up to 5 years, using point A as the reference point. This author found that the mean vertical growth was 4mm from the distance between point A and the nasion during the follow-up period, which was statistically significant (p < 0.001). It was not clear, however, which reference point was used for measuring changes in the position of point A. Based on a preliminary study by the same author, it may be inferred that a distance from that point to a 'perpendicular facial' line was used; there was also no description of which reference point was used to trace that perpendicular line.

Bradley et al.,^3^ although not mentioning growth specifically, found that the horizontal movement between the early and late postoperative period was always in the posterior direction (loss of results), for both the CMFA and DOM groups. There is no mention about a more anterior positioning of point A in the later postoperative period compared to the early postoperative period, from which it may be concluded that there was no sagittal growth in the series.

In a series consisting of 15 patients undergoing DOLF with an external distractor and monitored for one year, Shetye et al.^29^ found that anterior positioning of point A was 0.81mm in the late postoperative period, compared to early postoperative results. This was considered as “continued growth of the midface following distraction osteogenesis with a Le Fort III type osteotomy. In the same study, loss of results was 0.07mm (orbit point) and 1.34mm (margin of the upper incisor). Vertical facial growth was 0.95mm (point A) and 0.94mm (orbit point). However, in this study the true horizontal line was used as a parameter for measurements, thus repeating the possible failure in Kaban et al.'s^34^ and David and Sheen's method.^35^

In our study we found no horizontal gain for point A and the orbit point between the early and late postoperative period in any of the 11 patients. Values for vertical position alterations between the early and late postoperative period, which may be interpreted as facial growth, were 3.45mm (mean) and 3.4mm (median) for point A, and 2.92mm (mean) and 2.3mm (median) for point O.

## CONCLUSION

The procedures resulted in significant advancement. The horizontal component of skeletal movement predominated over the vertical component. The late posterior horizontal repositioning rate (loss of results) was minimal. Evident late postoperative vertical change in the position of reference points was observed compared to the early postoperative period; this was evidence of vertical facial growth. On the other hand, there was minor posterior repositioning and no evidence of growth in the horizontal direction.
